# Consensus-based cross-European recommendations for the identification, measurement and valuation of costs in health economic evaluations: a European Delphi study

**DOI:** 10.1007/s10198-017-0947-x

**Published:** 2017-12-19

**Authors:** Lisanne I. van Lier, Judith E. Bosmans, Hein P. J. van Hout, Lidwine B. Mokkink, Wilbert B. van den Hout, G. Ardine de Wit, Carmen D. Dirksen, Henk L. G. R. Nies, Cees M. P. M. Hertogh, Henriëtte G. van der Roest

**Affiliations:** 1 Department of General Practice and Elderly Care Medicine and Amsterdam Public Health Research Institute, VU University Medical Center, Van der Boechorststraat 7, room D-534, P.O. Box 7057, 1007 MB Amsterdam, The Netherlands; 2 Department of Health Sciences and Amsterdam Public Health Research Institute, Faculty of Earth and Life Sciences, Vrije Universiteit Amsterdam, Room U-430, De Boelelaan 1085, 1081 HV Amsterdam, The Netherlands; 3 Department of Epidemiology and Biostatistics and Amsterdam Public Health Research Institute, VU University Medical Center, De Boelelaan 1089a, 1081 HV Amsterdam, The Netherlands; 4 Department of Medical Decision Making and Quality of Care, Leiden University Medical Center, P.O. Box 9600, 2300 RC Leiden, The Netherlands; 5 Julius Center for Health Sciences and Primary Care, University Medical Center Utrecht, P.O. Box 85500, 3508 GA Utrecht, The Netherlands; 6 Department of Clinical Epidemiology and Medical Technology Assessment (KEMTA), Maastricht University Medical Centre, P.O. Box 5800, 6202 AZ Maastricht, The Netherlands; 7 Care and Public Health Research Institue, Maastricht University Medical Centre, P.O. Box 616, 6200 MD Maastricht, The Netherlands; 8 Vilans, P.O. Box 8228, 3503 RE Utrecht, The Netherlands; 9 Department of Organization Sciences, Faculty of Social Science, Vrije Universiteit Amsterdam, De Boelelaan 1081, 1081 HV Amsterdam, The Netherlands; 10 Department of General Practice and Amsterdam Public Health Research Institute, VU University Medical Center, Van der Boechorststraat 7, room B-546, P.O. Box 7057, 1007 MB Amsterdam, The Netherlands

**Keywords:** Delphi technique, Costing recommendations, Economic evaluation, Cross-country studies, I19

## Abstract

**Objectives:**

Differences between country-specific guidelines for economic evaluations complicate the execution of international economic evaluations. The aim of this study was to develop cross-European recommendations for the identification, measurement and valuation of resource use and lost productivity in economic evaluations using a Delphi procedure.

**Methods:**

A comprehensive literature search was conducted to identify European guidelines on the execution of economic evaluations or costing studies as part of economic evaluations. Guideline recommendations were extracted by two independent reviewers and formed the basis for the first round of the Delphi study, which was conducted among European health economic experts. During three written rounds, consensus (agreement of 67% or higher) was sought on items concerning the identification, measurement and valuation of costs.

**Results:**

Recommendations from 18 guidelines were extracted. Consensus among 26 panellists from 17 European countries was reached on 61 of 68 items. The recommendations from the Delphi study are to adopt a societal perspective, to use patient report for measuring resource use and lost productivity, to value both constructs with use of country-specific standardized/unit costs and to use country-specific discounting rates.

**Conclusion:**

This study provides consensus-based cross-European recommendations on how to measure and value resource use and lost productivity in economic evaluations. These recommendations are expected to support researchers, healthcare professionals, and policymakers in executing and appraising economic evaluations performed in international contexts.

**Electronic supplementary material:**

The online version of this article (10.1007/s10198-017-0947-x) contains supplementary material, which is available to authorized users.

## Introduction

The introduction of new pharmaceuticals and technologies has caused healthcare costs to steadily rise over recent decades in Europe [1, 2]. This threatens the sustainability of healthcare systems, and forces policymakers and financial stakeholders to make decisions on how to allocate scarce resources. Economic evaluations in which costs and effects of two or more healthcare interventions or innovations are compared can support decision makers in such allocation decisions [3]. Some countries have established cost-effectiveness as a decision criterion to reimburse healthcare interventions, especially for the reimbursement of new pharmaceuticals following their market approval [4].

To ensure the comparability and quality of economic evaluations, several European national agencies developed methodological guidelines on the principles and methods for the design, execution and reporting of economic evaluations over recent decades. In many countries, for example in Belgium, Germany, Norway, Portugal, Poland, the Netherlands, the Slovakia, and Slovenia [4–10], it is mandatory to prepare an economic evaluation in accordance with the national guidelines when applications are being made for reimbursement of a new healthcare technology.

In recent years, the EU stimulated the development of multidisciplinary partnerships among government agencies, research institutions and health ministries across European countries by funding several health technology assessment (HTA) projects at the European level [11–13]. Also, the number of international economic evaluations is growing, for example in situations where health interventions emerge at more or less the same time across different countries, or when it is not feasible to perform an economic evaluation with sufficient power in one country. An example is the European Schizophrenia Outpatient Health Outcomes (SOHO) study, in which the cost utility of treating schizophrenic patients with different types of antipsychotics was determined with use of data from ten countries [14]. Although effectiveness data from cross-country studies are often easily transferable to other settings, costing data are much more context specific. Despite the existence of many national guidelines, there is still little guidance on appropriate costing methods to use when health economic evaluations are conducted in international contexts, which hinders the comparability and transferability of results. Practical difficulties encountered when a cross-country study is performed include variation in the inclusion or exclusion of cost categories, in the classification of cost categories, in the choice of discount rate, and in the valuation of costs [4, 15]. Incomparability of international economic evaluations may result in unnecessary work and expenses, because researchers replicate economic evaluations to resemble their own specific context. Thus, to increase the comparability and transferability of economic evaluations in Europe, it is desirable to have a common set of detailed guidelines for the design and conduct of economic evaluations. The availability of such a set of guidelines will strengthen cross-border HTA collaborations such as those already existing within the European Network for Health Technology Assessment (EUnetHTA), but will also be useful for countries without country-specific guidelines for economic evaluations. Eliciting experts’ opinions on guidelines for economic evaluations will constitute an important step towards developing a common European view on conducting health economic evaluations. Therefore, the aim of this study was to develop cross-European recommendations for the identification, measurement and valuation of resource use and lost productivity for use in cross-European economic evaluations from a societal perspective, with use of a Delphi procedure among European health economic experts. A Delphi procedure was chosen because it is a structured approach to make group-based decisions on topics where strong differences in opinion exist. This method is commonly used to develop costing guidelines and reporting checklists for costing studies [16–20].

## Methods

This study is part of the European Identifying Best Practices for Care-Dependent Elderly by Benchmarking Costs and Outcomes of Community Care (IBenC) project. IBenC’s primary aim is to identify best practices of community care delivery systems across Europe by comparing their costs and quality of care outcomes while also developing methods to support the accomplishment of this aim [21].

### Study design

A Delphi study with three consecutive, blinded rounds was conducted between December 2014 and January 2015 among European health economic experts. The Delphi study was conducted online to ensure anonymity of the panellists. A steering committee was appointed to force consensus on items that were not agreed on by the panel after the final Delphi round [22–24].

### Delphi panel

A group of 110 international experts working in the field of health economics at government agencies, universities, research institutes and pharmaceutical agencies from 27 European countries was informed and invited by e-mail to participate in the Delphi study. Experts were selected on the basis of HTA-related publications in peer-reviewed journals, their participation in the development of national guidelines, their participation in other European HTA projects or their involvement with the International Society for Pharmacoeconomics and Outcomes Research (ISPOR) or EUnetHTA. Experts who were unable to participate in a Delphi round, but expressed their interest, were invited again for the subsequent round.

### Steering committee

An independent steering committee consisting of four professionals (WH, AW, CD and HN) in the field of health economics decided on items for which no consensus was reached after the third Delphi round. None of them were involved in selecting potential panellists, designing the questionnaires and analysing the results.

### Identification and review of existing guidelines

The questionnaire used in the first round of the Delphi study was based on a review of existing European guidelines for designing and conducting economic evaluations or costing studies as part of economic evaluations. To identify existing guidelines, the website of the ISPOR was searched [25]. ISPOR provides an overview of national pharmaceutical guidelines for economic evaluations. This overview is updated regularly on the basis of contacts with professionals in more than 50 countries to ensure its quality and accuracy. For countries for which no guideline was available on the ISPOR website, additional searches though the Internet were performed. Publicly available, English-language national guidelines containing recommendations on the execution of economic evaluations available issued by European government agencies were included. No exclusion criteria were applied, and the publication date was not restricted. Special effort was made to identify the most recent versions of the identified guidelines; webpages of HTA agencies were additionally searched, and authors of guidelines issued before 2003 were contacted for possible updates. A recent update of the Hungarian guideline was not available in English. Therefore, we consulted a health economist from Hungary to identify the most important differences between the two versions of the guideline. Every guideline was reviewed in detail, and information was extracted by two of the authors (LL and JB) independently. Discrepancies were discussed until consensus was reached. A standardized table to synthesize the recommendations was prepared a priori containing relevant issues: perspective; identification of resource use; measurement of resource use; valuation of resource use; discounting of future costs and discount rate used; incremental analysis of costs; sensitivity analysis; modelling; availability of a list with national standard unit costs [3, 26].

### Delphi study

The recommendations extracted from the identified guidelines were used to formulate questions for the first Delphi round. In this Delphi study, the starting point was the societal perspective for identifying relevant costs in an economic evaluation, because this is the most comprehensive perspective. Other commonly used perspectives, such as the healthcare and government perspectives, can be derived from this perspective. For each item, panellists were asked to indicate the most appropriate option, or to choose “no expertise” if they felt they had insufficient expertise on a specific topic. To capture recent methodological developments that were not included in the identified guidelines, alternative response options for each question could be provided by panellists. In all rounds, panellists were asked to justify their answers to every question. Together with the questions from the second and third rounds, panellists received a feedback report with the individual and group results from the previous round.

Consensus among the panel was defined a priori as an agreement of 67% or higher to include or exclude a specific item (i.e. a particular perspective, cost category, valuation or assessment method, discounting rate or study design) rated with a dichotomous response option. Agreement of 67% or higher is commonly used in Delphi studies to indicate consensus [27]. Agreement among the panel was calculated for each item separately in every Delphi round.

In the first round, we asked the Delphi panel to indicate for each of the listed perspectives, cost categories, and resource use items whether they should be included in an economic evaluation conducted from a societal perspective. Additionally, the panel was asked to indicate appropriate methods for measuring and valuing resource use and lost productivity, and whether value added taxes (VAT) should be included for all cost categories.

Questions in the second round were developed on the basis of the analysis of the previous responses. Items for which no consensus was reached in the first Delphi round were included once more, accompanied by arguments for and against inclusion reported in the first Delphi round. Alternative perspectives, new resource use items and alternative valuation methods suggested by the panellists in the first Delphi round were put forward for consideration as well. In addition, panellists were asked to rank the listed methods for assessing and valuing resource use and lost productivity with regard to their relevance. Finally, the panellists were asked to indicate which of the listed discounting rates and study design they found appropriate.

To construct the questionnaire for the third Delphi round, rankings on relevance given by the panellists in the second round were converted into relevance scores (a higher score indicates a more relevant method). First, for each respondent, the least relevant method was awarded one point, and the next relevant method was awarded one point more than the previous method. An exception was made for the two methods that were considered most relevant by the panellist. To discriminate better between methods ranked first and second and the other methods, the method placed in the second position received a relevance score twice that of the method in the third position. The method placed in the first position received twice the relevance score of the method in the second position. Subsequently, mean relevance scores for every method were calculated by summation of relevance scores and their division by the number of panellists. The final rankings were based on these mean relevance scores.

On the basis of the relevance scores, the two methods considered most relevant per topic were presented in the third Delphi round. Panellists were asked to choose the method they found most suitable to use in a European economic evaluation from a societal perspective. Also, items for which no consensus was reached in the second Delphi round were addressed again.

Finally, when no consensus was reached on an item, the steering committee was requested to make a final decision. By e-mail, the steering committee was provided with the arguments for and against a specific recommendation given by the panellists so that the members of the steering committee could weight these considerations to reach a final decision. Individual opinions from the members of the steering committee were gathered and used to make a final decision.

## Results

### Literature review

Eighteen national guidelines were included in the study. These guidelines were published in Austria, the Baltic states (Latvia, Estonia, Lithuania), Belgium, Croatia, Denmark, Finland, France, Germany, Hungary, Ireland, Italy, the Netherlands, Norway, Poland, Portugal, Spain, Sweden and the UK [5–10, 28–40]. For other European countries, no national guideline in English could be identified.

Table 1 provides a structured summary of the main recommendations from the identified guidelines. In short, six of the 18 identified guidelines recommend a societal perspective in the economic evaluation (the guideline from Portugal recommends in addition the use of a third-party payer perspective), six recommend a healthcare perspective and another six recommend both perspectives. Most guidelines stated clearly which costs should be included in a health economic evaluation. All guidelines stressed that all relevant direct healthcare costs should be included for which differences are expected between treatments. The inclusion of social care costs such as those resulting from the use of respite care and supportive care services was recommended in ten guidelines, the inclusion of patient and family costs, including patient out-of-pocket expenses, time costs, informal care costs and travel costs, was recommended in 12 guidelines and the inclusion of lost productivity costs was recommended in 11 guidelines. Five of the 18 guidelines did not describe which sources should preferably be used for the measurement of resource use/costs. There was large variation between the guidelines with regard to the valuation of the resources used. Seven of the 18 guidelines stressed that the valuation method chosen should reflect the opportunity costs (i.e. the value of the forgone benefits because the resource is not available for its best alternative use). In the other guidelines, the underlying principle for valuation was not described. Various valuation methods were recommended in the guidelines, including the use of standard unit costs, tariffs, lowest price, diagnosis-related groups and macro costing.

**Table 1:** Overview of country-specific recommendations regarding perspective, identification of costs, measurement of costs, valuation of costs, discounting, type of economic evaluation, and availability of a list with country-specific unit costs

Country	Perspective	Identification of costs	Measurement of costs	Valuation of costs	Discounting of future costs	List of unit costs available
Austria [28]	Societal	Healthcare, productivity	Patient level, primary level, secondary level	Opportunity costs, market prices, shadow prices, human capital method	5% SA, higher and lower rates (e.g. 3% and 10%)	No
Baltic states: Latvia, Estonia, Lithuania [29]	Societal, healthcare	Healthcare, social care, patient and family	–	Tariffs	5%	No
Belgium [5]	Healthcare	Healthcare	Patient level, primary level, secondary level	Opportunity costs, unit costs	3% SA, not specified	Yes
Croatia [30]	Societal, healthcare	Healthcare	–	Tariffs	5% SA, 3–10%	No
Denmark [31]	Societal	Healthcare, social care, patient and family, productivity, future costs	Patient level, primary level, secondary level, expert opinion	Opportunity costs, unit costs	–	No
Finland [32]	Healthcare	Healthcare, social care, patient and family, productivity	Patient level, primary level	Unit costs, tariffs	3%	No
France [33]	Healthcare	Healthcare, social care, patient and family	Patient level, primary level, secondary level	Opportunity costs, tariffs	4% SA, not specified	No
Germany [6]	Healthcare	Healthcare, social care, productivity	Patient level, primary level	Macro costing, micro costing, diagnosis-related group, human capital method	3% SA, 0%, 5%, 7% and 10%	No
Hungary [34, 35]	Societal, healthcare	Healthcare, patient and family, productivity	Patient level, primary level	Tariffs, lowest price, include VAT	3.7% SA, 3–6%	Yes
Ireland [36]	Healthcare	Healthcare	Patient level, primary level, secondary level, clinical practice guidelines, expert opinion	Micro costing, macro costing	4% SA, 0–6%	No
Italy [37]	Societal, healthcare	Healthcare, patient and family, productivity	Patient level	Opportunity costs, micro costing, human capital method	3 and 0% SA, 0–8%	No
The Netherlands [10]	Societal	Healthcare, social care, patient and family, productivity	Patient level, primary level	Unit costs, standard costs, cost price calculation, friction cost method	4% SA, not specified	Yes
Norway [7]	Societal	Healthcare, patient and family, productivity	–	Market prices, exclude VAT	4%	Unclear
Poland [9]	Societal, healthcare	Healthcare, patient and family, productivity	Patient level, primary level, secondary level	Unit costs, standard costs, tariffs, cost price calculation	5% SA, 0%	No
Portugal [8]	Societal, 3rd-party payer	Healthcare, social care, patient and family, productivity	Patient level, primary level, secondary level	Opportunity costs, unit costs	5% SA, not specified	No
Spain [38]	Societal, healthcare	Healthcare, social care, patient and family, productivity	Patient level, primary level	Opportunity costs, unit costs	3% SA, 0% and 5%	No
Sweden [39]	Societal	Healthcare, social care, patient and family, productivity	–	Human capital method, unit costs	3% SA, 0% and 5%	Only medication
UK [40]	Healthcare	Healthcare, social care	–	Tariffs	3.5% SA, 1.5%	No

*SA* sensitivity analysis, *VAT* value added tax

### Delphi panel

Of the 110 invited experts, 26 (24%) participated in one or more rounds, six agreed to participate but did not participate (5%), 48 (44%) did not respond and the remainder (30, 27%) did not wish to participate mainly because of time constraints. Of the 26 invitees who participated, 11 (42%) participated in all three rounds, three (12%) participated in two rounds, and 12 (46%) participated in one round. Each round was completed by at least 16 experts. Background information on the panellists is presented in Table 2.

**Table 2:** Characteristics of the Delphi panel: country of residence, primary employment and mean number of years of experience in health technology assessment (HTA)

Characteristics	Delphi round 1 (*n* = 19)	Delphi round 2 (*n* = 16)	Delphi round 3 (*n* = 16)
Country of residence	*n* = 1: Austria, Bulgaria, Croatia, Cyprus, Czech Republic, France, Germany, Ireland, Italy, Lithuania, Poland, Slovakia, Slovenia, Spain. *n* = 2: Sweden. *n* = 3: The Netherlands	*n* = 1: Austria, Bulgaria, Cyprus, Czech Republic, France, Germany, Italy, Lithuania, Poland, Slovakia, Slovenia, Spain, Sweden, UK. *n* = 2: The Netherlands	*n* = 1: Bulgaria, Croatia, Cyprus, France, Germany, Lithuania, Poland, Slovakia, Slovenia, Spain, Sweden, UK. *n* = 2: Austria, The Netherlands
Primary employment			
University	9 (47%)	5 (31%)	4 (25%)
Government institution	6 (31%)	7 (44%)	9 (57%)
Healthcare and/or research institute	2 (11%)	2 (13%)	1 (6%)
Pharmaceutical company	–	1 (6%)	1 (6%)
Consulting company	2 (11%)	1 (6%)	1 (6%)
Mean number of years of experience in HTA	14.1 (range 1–40)	12.4 (range 1–40)	10.5 (range 1–16)

### Delphi results

Table 3 presents a structured summary of the Delphi results. It includes the agreement (%) per item among the panel in the three Delphi rounds and among the steering committee, the final ranking of the methods for measuring and valuing resource use and lost productivity, and the choice for most suitable method.

**Table 3:** Results of the Delphi study: perspective, inclusion of cost categories and value added tax (VAT), measurement and valuation of resource use and lost productivity, discounting, type of economic evaluation, and study design

Component/topic	Delphi round 1 (*n* = 19)	Delphi round 2 (*n* = 16)	Delphi round 3 (*n* = 16)	Steering committee (*n* = 4)
	Inclusion	Inclusion	Inclusion	Inclusion
Perspective				
Healthcare sector	**96%**			
Societal	**88%**			
Government^a^		40%		
Healthcare payer(s)^a^		**86%**		
Health insurance–public funds^a^		**87%**		
Social services^a^		**67%**		
Identification of costs (societal perspective)				
1. Healthcare services				
Hospitalization; ICU; emergency visits; medical specialist at an outpatient clinic; diagnostic services; medical devices; treatment procedures; day treatment in a hospital; medication; allied healthcare providers; mental healthcare services; preventive care; general practitioner visits; institutionalized care; palliative care; home care	**94–100%**			
Supportive care; social care/welfare; respite care	**73–89%**			
Complementary therapists	50%	63%		**75%**
E-health^a^		**71%**		
2. Intervention costs				
Administration; planning; implementation; supervision and monitoring	**67–80%**			
Development	50%	40%		*25*%
Training	60%	60%		**100%**
Donated items (such as drugs, vaccines, supplies or equipment)	60%	**71%**		
3. Patient and family costs				
Patient-out-of-pocket expenses; patient time; travel costs; informal caregivers time (not fully compensated); informal caregivers time (fully compensated)	**76–94%**			
4. Lost productivity costs				
Absenteeism; reduced productivity while at work (i.e. presenteeism)	**83–100%**			
Absenteeism from unpaid labour such as household activities, education, voluntary work	44%	*27%*		
5. Future costs				
Future healthcare costs incurred for disorders related to the intervention	**100%**			
Future healthcare costs incurred for disorders unrelated to the intervention	53%	50%		**100%**
Future non-healthcare expenditures (e.g. food, clothes, and housing)	39%	*19%*		
VAT				
Including VAT	50%	**79%**		

	Inclusion	Panel ranking	Most suitable	Most suitable
Measurement of resource use				
1. Healthcare services				
Patient-level data: patient-based reports (resource use questionnaires and interviews, self-reported activity logs, cost diaries etc.)	**94%**	**1**	50%	**75%**
Patient-level data: observer/care provider-based reports (medical records, time and motion records, etc.)	**94%**	3		
Secondary-level data: local registers	**89%**	6		
Secondary-level data: national registers	**89%**	4		
Secondary-level data: national insurance fund utilization databases	**89%**	**2**	50%	*25%*
Secondary-level data: hospital information system	**89%**	5		
Estimates based on clinical practice guidelines	**78%**	7		
Expert opinion	**67%**	8		
2. Travel costs				
Standard distances	**100%**	**1**	**100%**	
Patient-reported distances	65%	**2**		
Public transport should be valued by market prices and travelling by car using standard costs per kilometre/mile^a^		86%		
3. Absenteeism from paid labour				
Company registered data for sick leave	**81%**	**1**	50%	0%
Self-reported sick leave due to the disease under study	**81%**	**2**	50%	**100%**
Self-reported sick leave due to general health	63%	4	–	–
Use of published estimates of previous studies	**71%**	3	–	–
4. Presenteeism				
Self-reported perceived performance during working hours due to the disease under study	**80%**	**2**	*27%*	–
Self-reported perceived performance during working hours due to general health	47%	4		
Self-reported comparative performance (how an employee’s performance differs from that of others or from his/her usual performance)	40%	3		
Self-reported rating of both the quantity and quality of the work (quantity and quality method)	**79%**	**1**	**73%**	
Self-reported unproductive time while at work	**67%**	5		
Valuation of resource use				
1. Unit costs to value healthcare utilization				
Average of available European unit costs	*29%*			
Lowest available of European unit costs	*12%*			
Highest available of European unit costs	*12%*			
Use of costs from 1 or more other countries and their conversion with use of power purchasing parities	47%	**2**		
Country-specific unit costs	**100%**	**1**	**100%**	
2. Healthcare services				
Standard/unit costs	**100%**	**1**	*23%*	
Market prices	60%	4		
Tariffs	46%	6		
Bottom-up/micro costing estimation of unit costs	**87%**	**3**		
Top-down/macro costing estimation of unit costs	40%	7		
Diagnosis-related groups (payment weight based on the average resources used to treat patients in that diagnosis-related group)	**75%**	5		
Country-specific standardized values^a^	–	**2**	**77%**	
3. Supportive care/social care services				
Standard/unit costs	**94%**	**1**	*23%*	
Market prices	**69%**	4		
Tariffs	40%	5		
Bottom-up cost price calculation	**81%**	**3**		
Top-down cost price calculation	44%	6		
Country-specific standardized values^a^		**2**	**77%**	
4. Patient out-of-pocket expenses				
Patient-reported costs	**75%**	**1**	**75%**	
Standard/unit costs	**81%**	**2**	*25%*	
Market prices	**75%**	3		
Tariffs	47%	5		
Bottom-up cost price calculation	**69%**	4		
Top-down cost price calculation	44%	6		
5. Patient time/informal care				
National average wages of unskilled labour	59%	3		
National average wages of unskilled labour sex/age specific	44%	**2**	46%	50%
National minimum wages of the population as a whole	*25%*			
National minimum wages of the population as a whole sex/age specific	*31%*			
Specific (self-reported) wages	35%	4		
Shadow prices (opportunity costs when the actual price is not known or difficult to calculate)	**73%**	**1**	54%	50%
National average wages to reflect the value of leisure time^a^		5		
6. Travel costs				
Patient-reported costs	61%	**2**		
Standard/unit costs	**94%**	**1**		
Market prices	**76%**	3		
7. Absenteeism (1) approach				
Friction cost approach	**82%**	**1**	**73%**	
Human capital approach	59%	**2**	*27%*	
8. Absenteeism (2) proxy measure				
National average wages of unskilled labour	*31%*			
National average wages of unskilled labour sex/age specific	*31%*			
National average wages of the population as a whole	**65%**	**2**	*7%*	
National average wages of the population as a whole sex/age specific	**71%**	**1**	**93%**	
Specific (self-reported) wages	**63%**	3		
National minimum wages	*19%*			
9. Presenteeism				
National average wages of unskilled labour	*27%*			
National average wages of unskilled labour sex/age specific	*27%*			
National average wages of the population as a whole	60%	**2**	*8%*	
National average wages of the population as a whole sex/age specific	**67%**	**1**	**92%**	
Specific (self-reported) wages	53%	3		
National minimum wages	*13%*			
10. Unpaid labour				
National average wages of unskilled labour	47%	**2**		
National average wages of unskilled labour sex/age specific	47%	3		
National average wages of the population as a whole	*29%*			
National average wages of the population as a whole sex/age specific	41%	5		
Specific (self-reported) wages	53%	4		
Shadow prices (opportunity costs when the actual price is not known or difficult to calculate)	**73%**	**1**		
National minimum wages	*29%*			

	–	Inclusion	Most suitable	Inclusion
Discounting				
European average discount rate		54%		
Lowest European discount rate		*8%*		
Highest European discount rate		*8%*		
Country-specific discount rate		**80%**		
Study design				
Model based (outcomes of an average patient are assessed)		**86%**		
Trial based (outcomes of an individual patient are assessed)		**86%**		

^a^ New item suggested by participant(s) in the previous Delphi round

Round 1: Agreement (%) on perspective, cost items and inclusion of VAT, addition of new items, identification of appropriate methods to measure and value resource use and lost productivity. Round 2: Agreement on new items and readdressing items with lack of consensus, ranking identified methods on relevance, and agreement (%) on inclusion of discounting, and study design. Round 3: Identification of the most suitable method per category of the two highest ranked methods in round 2. Steering committee: Final decision on items lacking consensus. Agreement is given in bold when consensus for inclusion was considered (agreement of 67% or higher), and in italic when the panel agreed that an item should not be included (agreement for exclusion of 67% or higher)

The panel reached consensus on 58 of 65 items (89%). The steering committee decided on the seven items without consensus after three rounds. These items comprised the inclusion of four cost items, two measurement methods and one valuation method.

### Recommendations

Table 4 gives an overview of the consensus-based recommendations that were developed on the basis of the results of the Delphi study. The results and supporting comments from the panellists are addressed below.

**Table 4:** Summary of consensus-based recommendations

Component/topic	Recommendation	Arguments
Perspective	Societal perspective	It is likely that relevant costs are missed when a narrower perspective is used, because often sectors other than healthcare may incur costs or costs savings as a result of the intervention
Identification of costs	Depending on the nature of the disorder, intervention and treatment under study. Costs of healthcare services and social care services, intervention costs, patient and family costs, lost productivity costs, and future healthcare costs (See Appendix 1 in the electronic supplementary material for more details)	Relevant cost categories
Measurement of resource use		
Healthcare services	Patient-level data	Patient self-report methods are preferred over national databases because not all services are covered in these databases
Patient-out-of-pocket expenses	Patient-reported expenses	Most reliable source to obtain these data
Patient time costs	Patient-reported time	Most reliable source to obtain these data
Travel costs	Standard distances	To avoid random differences between groups
Informal care costs	Self-report of informal caregivers	Most reliable source to obtain these data
Absenteeism from paid labour	Self-reported sick leave due to the disease under study	Can be more accurately attributed to the disorder under study and may more easily be available than reports from many individual employers
Presenteeism	Obtain ratings of both the quantity and the quality of work performed in a standardized way	Standardized method
Valuation of resource use		
Healthcare services	Country-specific standard/unit costs	Representative of the situation in the country under study
Supportive care/social care services	Country-specific standard/unit costs	Representative of the situation in the country under study
Patient out-of- pocket expenses	Patient-reported costs	Large variations between patients
Patient time/informal care	National average wages of unskilled labour sex/age specific	
Travel costs	Use of standard distances between the patient’s home and the healthcare provider Travel by public transport: tariffs Travel by car: standard costs per kilometre/mile	Tariffs are closely related to market prices, and are expected to resemble opportunity costs adequately Standard costs per kilometre/mile to avoid random differences between groups
Absenteeism	Friction cost approach. National sex/age-specific average wages of the population as a whole	Human capital approach, an alternative, is expected to lead to overestimation of productivity losses
Presenteeism	National sex/age-specific average wages of the population as a whole	
VAT	Include VAT	Part of the true costs of healthcare
Discounting	Country-specific discounting rates Sensitivity analysis: lowest and highest European discounting rates	Representative of the situation in the country under study
Study design	Both a model-based approach and a trial-based approach are appropriate depending on the research question under study	

*VAT* value added taxes

### Perspective

The societal perspective is recommended for economic evaluations in a cross-European context (88% agreement). The societal perspective helps to identify cost shifting between sectors. According to the panel, it is likely that relevant costs are missed when a narrower perspective is used because often sectors other than healthcare may incur costs or costs savings as a result of the intervention.

### Identification of resource use and lost productivity

It is recommended to take a broad perspective with regard to costs, and to include all cost categories for which differences are expected between treatments. The relevant cost categories according to the Delphi panel are healthcare service costs, intervention costs, patient and family costs, lost productivity costs and future costs.

Healthcare services are services directly related to the prevention, diagnosis, treatment, rehabilitation and nursing care for a particular disorder. Assistance with (instrumental) activities of daily living also falls into this category. The panellists did not reach consensus on the inclusion of costs of complementary therapies (round 1, 50% agreement; round 2, 63% agreement). The arguments provided by the panellists in favour of inclusion were that all related resource use should be included, and that costs of complementary therapies may be significant. A counterargument was that complementary therapists do not represent evidence-based medicine. The steering committee recommended that these costs should be included, since these costs represent resources that were used. Depending on the country’s funding system, complementary therapies may be reimbursed by health insurance and are then considered part of healthcare costs. If the costs are not reimbursed, they have to be categorized as out-of-pocket costs. An additional category that emerged from the Delphi study was the costs of e-health interventions (round 2, 71% agreement).

Intervention costs include all costs related to the implementation of a particular intervention, and should cover both personnel costs [time needed for administration (75% agreement), planning (69% agreement), implementation (67% agreement), and supervision and monitoring (80% agreement)], and the costs of materials needed for implementation of the intervention, including costs of donated items (such as drugs, vaccines, supplies or equipment) (71% agreement). On the inclusion of development and training costs of the intervention, no consensus was reached (round 1, 50% and 60% agreement, respectively; round 2, 40% and 60% agreement, respectively). The panellists’ arguments in favour of inclusion were that all production costs should be included, ad that training costs should be included if training is a prerequisite for the intervention to be effective. However, some argued against inclusion, because a ‘steady state’ should be assumed; the cost of development is usually covered by the price of the intervention. The steering committee recommended that a ‘steady state’ of the intervention should be assumed, implying that costs are estimated for routine implementation of the intervention in daily practice, and thus not to include development costs separately. If training of staff is a prerequisite for adequate execution of the intervention, then training costs represent a true use of resources and should be included. However, if only a one-time initial training is required, it is recommended not to include these costs. Care should be taken that intervention costs are not double counted with healthcare costs.

Patient and family costs include expenses incurred by patients as a consequence of the disorder under study, and costs of informal care. The following cost categories should be taken into account: patient-out-of-pocket expenses such as costs of over-the-counter-medication and costs of assistive aids (89% agreement); patient time costs associated with seeking and receiving care for the disorder under study (78% agreement); the costs travel required by patients (84% agreement); and informal care costs (94% agreement). Informal care costs include costs related to time spent and resources used by informal caregivers that are not compensated (76% agreement) or not partially or fully compensated (94% agreement).

Lost productivity costs are defined as costs related to reduced productivity from paid labour as a direct consequence of the disorder under study. The costs of both absenteeism (i.e. absence from paid and unpaid work) (100% agreement) and presenteeism (i.e. reduced efficiency when present at work) (83% agreement) should be included in an economic evaluation from a societal perspective. Lost productivity costs due to absenteeism from unpaid labour should not be included in an economic evaluation according to the panel (73% agreement). The main argument not to include these costs was the lack of standardized methods to value unpaid labour, leading to unreliable cost estimates and a risk of double counting. However, it was also argued that costs of absenteeism from unpaid labour may be an important cost category in specific patient populations such as elderly people, where the proportion of people performing unpaid labour is higher as compared with the general population.

Future healthcare costs are costs for treatment of disorders occurring in life years gained as a result of the intervention under study. Part of the future healthcare costs is directly related to the intervention, whereas other costs are not (e.g. costs for dementia treatment in added years because of successful cancer treatment). Future healthcare costs related to the intervention should be included according to the panel (100% agreement). A consensus was not reached on the inclusion of healthcare costs unrelated to the intervention (round 1, 53% agreement; round 2, 50% agreement). Panellists in favour of inclusion argued that unrelated future healthcare costs should theoretically be included if an intervention (significantly) prolongs life and if important differences in future costs between interventions are expected. However, others argued against inclusion on the basis that the calculations are difficult as many assumptions are made. The steering committee recommended the inclusion of related and unrelated future healthcare costs if the intervention is expected to result in an extension of life, because it represent a true use of resources.

A summary of relevant cost items per cost category is provided in Appendix 1 in the electronic supplementary material.

### Measurement of resource use and lost productivity

The panellists did not agree on the most suitable method to assess healthcare utilization and were divided between patient-based reporting and the use of national insurance fund utilization databases (secondary-level data). Some panellists indicated that these databases are more accurate than patient-level data, whereas others argued that these databases are less precise, may not contain all relevant information and may create problems when data are linked to individual patients. The steering committee opted for patient-based reports as the most preferable method, because not all services are covered in national databases and such databases are not easily available for all countries.

The recommended methods to collect patient-reported data are resource use questionnaires and interviews, activity logs and cost diaries. If patients are incapable of self-reporting, proxy reports are recommended. Patient out-of-pocket expenses are country specific as they depend on the reimbursement level and funding system. Therefore, the patient is considered the most reliable source to obtain these data. Patient time costs are also preferably measured with use of patient reports. In situations where patient reports cannot be used, these costs can also be based on standard time estimates associated with seeking and receiving care. However, researchers should be aware that patient time costs in seeking care may overlap with absenteeism from paid labour, with the risk of double counting. When travel costs are being assessed, it is recommended to use standard distances from the patient’s home to the healthcare provider over patient-reported distances, to avoid random differences between groups (100% agreement). Costs related to time spent and resources used by informal caregivers should be based on self-reports.

No consensus was reached on what is considered the most suitable method to measure absenteeism from paid work (company-registered data or self-reported sick leave). The panellists in favour of using company registered data (50% of the panel) indicated that they are cheaper, easier to collect, more trustful and more credible than self-reported sick leave, whereas opponents (50%) argued that company-registered data are less precise, rarely available, and difficult to obtain in certain situations. The steering committee recommended that absenteeism from paid work should preferably be measured with self-report questionnaires, because self-reported productivity losses can be more accurately attributed to the disorder under study. Also, self-reported productivity losses may be more easily available than reports from a large number of different employers. The recommended method to collect data on presenteeism is to obtain ratings of both the quantity and the quality of work from the patient by standardized questionnaires (73% agreement).

### Valuation of resource use and lost productivity

Opportunity costs are recommended to value resource use. Costs used in an economic evaluation should be representative of the country under study. Therefore, country-specific costs are preferred (100% agreement), and European average costs should not be used (71% agreement). Since country-specific costs may differ considerably, resource use rates and costs should be presented separately to facilitate the generalization of study results to other settings.

The preferred proxy measure for the opportunity cost of healthcare services is country-specific standard unit costs, when available (77% agreement). Standard unit costs include all costs related to the provision of a particular service. However, standard unit cost estimates should not be used for patient out-of-pocket expenses because of large variations between patients (75% agreement). The panellists did not agree on the most suitable valuation method for the opportunity costs of patient time and informal care (shadow prices or national average wages of unskilled labour sex/age specific, 54% and 46%, respectively). The arguments in favour of the use of national averages wages were the availability and “otherwise disease affecting highly skilled or paid people have an advantage”, whereas the counterargument was that patients are not unskilled. The steering committee recommended the use of sex- and age-specific average wages of unskilled labour for the valuation of patient time and informal care, although the committee recognizes this may underestimate the true opportunity costs associated with informal care. Use of shadow prices may be more flexible since they can be adapted to the specific informal care situation. With regard to costs of traveling by public transport, tariffs should be used (86% agreement). Tariffs are expected to be closely related to market prices, and are therefore considered to resemble opportunity costs adequately. For travel by car, it is recommended to use standard costs per kilometre/mile, including costs for petrol, maintenance, depreciation, and taxes (86% agreement). Lost productivity should be valued with age- and sex-specific national average wages (93% agreement). For the valuation of absenteeism from paid work, the friction cost approach is preferred over the human capital approach because the latter is expected to lead to overestimation of productivity losses (73% agreement). With the friction cost approach, the period over which the production loss is calculated is limited to the friction period (i.e. the time that an employer needs to replace a sick employee). It should be taken into account that the length of the friction period depends on the local economic situation and that friction periods are not available for most European countries. Therefore, countries should try to determine the friction period for their country or, when this is not feasible, perform a sensitivity analysis in which productivity losses are valued with use of the human capital approach.

### Inclusion of VAT

VAT should preferably be included in the societal costs (79% agreement). VAT are indirect taxes on the domestic consumption of goods and services. Some goods are zero rated such as essential drugs and medical devices. Although VAT is a transfer from the individual to the government, it can be seen as part of the true costs of healthcare services according to the panel.

### Discounting

Country-specific discounting rates should be used in the reference case (80% agreement). It is recommended to perform sensitivity analyses with the lowest and highest European discounting rates.

### Study design

The choice of a model-based approach or a trial-based approach should depend on the research question according to the panellists. The study should be model based when the intervention effects are expected in the long term, when it is not possible to include all relevant treatment alternatives in one study, or when the incidence of the clinical end point is low. In other situations, a trial-based approach suffices.

## Discussion

This Delphi study aimed to develop cross-European recommendations for identifying, measuring and valuing resource use and productivity losses in economic evaluations. The use of a societal perspective is recommended for economic evaluations in a cross-European context because this perspective provides a broad view of the impact of healthcare interventions on society as a whole. Resource use is preferably measured by means of patient-reported measures and valued with use of country-specific standardized costs. Lost productivity is preferably measured by means of self-report and valued with use of the friction cost approach with age- and sex-specific national average wages.

The Delphi design used allowed us to elicit the opinion of a heterogeneous international panel of experts with extensive expertise in economic evaluations within a relatively short time. By inviting the panel to suggest more suitable, alternative options in addition to the ones prespecified, we expected to capture recent developments in this field as well. Consensus was reached relatively easily for items regarding identification of cost categories. It was more difficult to reach consensus for items regarding assessment and valuation of resource use and lost productivity: in Delphi round 1, no consensus was reached for 21 the 52 valuation methods (40%) that were listed in the questionnaire. This is in line with previous studies that tried to harmonize existing national guidelines [4, 15].

Recently, EUnetHTA developed a guideline with a framework for the method of economic evaluations based on common denominators in existing national guidelines from 25 European countries. The recommendations from our study on how to measure and value resource use and lost productivity in economic evaluations complement the EUnetHTA guideline since it does not extensively cover these topics. Also, the recommendations on topics discussed in both guidelines are in line with each other. Together, the EUnetHTA guideline and the recommendations described in this article are expected to support researchers, healthcare professionals and policymakers when they are conducting and appraising economic evaluations.

### Considerations

Because of its comprehensiveness, the societal perspective is recommended as the reference case. However, in some countries (e.g. Belgium, Italy, Germany, and the UK) decision making is solely based on the healthcare perspective. To provide relevant information for these specific countries, an additional analysis from a healthcare perspective is required. If indicated, additional sensitivity analyses can be performed from other relevant perspectives [e.g. health insurance–public funds or healthcare payer(s)]. From a societal perspective, prices should reflect societal opportunity costs. However, in some countries such prices are unavailable or may be closer to costs from the healthcare perspective, whereas other countries, such as the Netherlands, attempt to approach societal opportunity costs by providing standard costs. The Delphi panel was not asked to decide between discounted and undiscounted costs. However, to facilitate the transfer of the results to other settings, we suggest that both discounted and undiscounted costs are presented.

The lack of agreement among the panellists on the method to assess healthcare utilization may suggest that this is one area that it may not be possible to standardize, since national insurance fund databases differ considerably. On the basis of our Delphi study, the use of patient-based reports is recommended, but we are aware that may be infeasible in some economic evaluations, especially those that use a modelling approach.

Because of the lack of standardized methods to measure absenteeism from unpaid labour, these costs should not be included in an economic evaluation according to the panel. However, in some populations this may be an important cost category (e.g. elderly people). Therefore, further research is needed to develop appropriate methods to validly assess costs due to decreased productivity in unpaid work.

Among the panel and in the literature, strong differences of opinion exist with regard to the inclusion of unrelated future healthcare costs [41]. Inclusion or exclusion of these costs may have important distributional consequences for life-saving or life-extending interventions [42]. Thus, ignoring these costs may lead to suboptimal decisions [41]. Therefore, the steering committee decided to include these costs. Practical objections against inclusion can be tackled, at least for some part, as methods to accurately estimate unrelated future healthcare costs are being developed increasingly [42], although these methods are currently available for only a few EU counties. Further improvements in this area are needed.

### Limitations and strengths

Some limitations of the current study need to be acknowledged. First, the Delphi study had a lower response rate than anticipated; only one quarter of the invited experts participated in at least one of the Delphi rounds. This may have led to selection bias. However, since the panellists were distributed across Europe, the results likely reflect the variety in opinions and methods applied across Europe. Moreover, we considered the number of panellists to be sufficient, since the literature generally recommends 10–18 experts for a Delphi panel [16]. Second, the decisions of the steering committee might be dominated by the Dutch perspective, because all members were from the Netherlands. However, the steering committee members took into account the panellists’ arguments when making decisions, and cross-national expertise on funding systems was available in the steering committee. Finally, the recommendations are broadly formulated. This means that potentially relevant items in economic evaluations among specific patient populations are not included in this article. However, this can also be considered a strength of the study since this makes the guidelines applicable to a broad range of situations.

Other strengths are the structured and systematic approach used to identify agreement and disagreement in the methods among the panel and to generate consensus about the recommendations presented in this article. By developing recommendations based on existing guidelines and opinions of experts from all over Europe, we expect that the recommendations can be validly used across Europe. Also, the inclusion of generally accepted methods for economic evaluation greatly enhances the feasibility of complying with the recommendations formulated in this articles.

## Conclusion

We developed consensus-based recommendations for the identification, measurement and valuation of healthcare utilization and lost productivity that are applicable for European economic evaluations, despite differences between national guidelines. We think that these recommendations are generally acceptable because the recommendations were developed on the basis of a Delphi study among European experts in this field, and consensus was reached for most items within three rounds. Application of these recommendations will improve the comparability of economic evaluations conducted in Europe. Although pricing and reimbursement decisions will remain the full competence of national authorities, the recommendations will help decision makers better interpret the results of a study conducted elsewhere and facilitate the exchange of results between European countries. Moreover, it will allow countries to build on each other’s expertise and make cross-country evaluations easier to perform.

## Electronic supplementary material

Below is the link to the electronic supplementary material.

Supplementary material 1 (DOCX 24 kb)

## References

[1] Organisation for Economic Co-operation and Development: projecting OECD health and long-term care expenditures. What are the main drivers? Economics Department working papers no. 477. Organisation for Economic Co-operation and Development, Paris (2006)

[2] World Health Organization: The European health report 2009: health and health systems. WHO Regional Office for Europe, Copenhagen (2010)

[3] Drummond MF, Sculpher MJ, Torrance GW, O’Brien BJ, Stoddart GL (2005). Methods for the economic evaluation of health care programmes.

[4] Hjelmgren J, Berggren F, Andersson F (2001). Health economic guidelines—similarities, differences and some implications. Value Heal..

[5] Cleemput, I., Neyt, M., Sande, S. Van De, Thiry, N.: Belgische richtlijnen voor economische evaluaties en budget impact analyses: tweede editie. Health Technology Assessment (HTA). KCE Report 183A. D/2012/10.273/52. (2012)

[6] Institute for Quality and Efficiency in Health Care : General methods for the assessment of the relation of benefits to costs. Institute for Quality and Efficiency in Health Care, Cologne (2009)

[7] Norwegian Medicines Agency: Guidelines on how to conduct pharmacoeconomic analyses. Norwegian Medicines Agency, Oslo (2012)

[8] Da Silva EA, Pinto CG, Sampaio C, Pereira JA, Drummond M, Trindade R (1998). Guidelines for economic drug evaluation studies. INFARMED.

[9] Agency for Health Technology Assessment: Guidelines for Conducting health technology assessment (HTA). Agency for Health Technology Assessment, Warsaw (2009)

[10] Hakkaart-van Roijen, L., Tan, S.S., Bouwmans, C.A.M.: Handleiding voor kostenonderzoek, methoden en standaard kostprijzen voor economische evaluaties in de gezondheidszorg. College voor Zorgverzekeringen, Diemen (2010)

[11] Granados A, Jonsson E, Banta HD, Bero L, Bonair A, Cochet C (1997). EUR-ASSESS project subgroup report on dissemination and impact. Int. J. Technol. Assess. Health Care.

[12] Banta D, Oortwijn W (2001). Health technology assessment and health care in the European Union. TA-Datenbank-Nachrichten..

[13] Kristensen FB, Makela M, Neikter SA, Rehnqvist N, Haheim LL, Morland B (2009). European network for health technology assessment, EUnetHTA: planning, development, and implementation of a sustainable European network for health technology assessment. Int J Technol Assess Heal Care..

[14] Knapp M, Windmeijer F, Brown J, Kontodimas S, Tzivelekis S (2008). Cost-utility analysis of treatment with olanzapine compared with other antipsychotic treatments in patients with schizophrenia in the panEuropean SOHO study. PharmacoEconomics..

[15] Mathes T, Jacobs E, Morfeld JC, Pieper D (2013). Methods of international health technology assessment agencies for economic evaluations—a comparative analysis. BMC Health Serv Res..

[16] Okoli C, Pawlowski SD (2004). The delphi method as a research tool: an example design considerations and applications. Inf Manag..

[17] Linstone, H.A., Turoff, M.: Introduction. In: Linstone, H.A., Turoff, M. (eds.) The Delphi Method: Techniques and Applications. Addison-Wesley, Boston (1975)

[18] Ruger JR, Reiff MA (2016). A checklist for the conduct, reporting, and appraisal of microcosting studies in health care: protocol development. JMIR Res Protoc..

[19] Husereau D, Drummond M, Petrou S, Carswell C, Moher D, Greenberg D (2013). ISPOR health economic evaluation publication guidelines-CHEERS good reporting practices task force: consolidated health economic evaluation reporting standards (CHEERS)–explanation and elaboration: a report of the ISPOR health economic evaluation publication guidelines good reporting practices task force. Value Health..

[20] Uegaki K, de Bruijne MC, Anema JR, van der Beek AJ, van Tulder MW, van Mechelen W (2007). Consensus-based findings and recommendations for estimating the costs of health-related productivity loss from a company’s perspective. Scand. J. Work Environ. Health.

[21] Identifying best practices for care-dependent elderly by benchmarking costs and outcomes of community care (IBenC): https://www.ibenc.eu/ (2017). Accessed 2 Jan 2017

[22] Mokkink LB, Terwee CB, Patrick DL, Alonso J, Stratford PW, Knol DL (2010). International consensus on taxonomy, terminology, and definitions of measurement properties for health-related patient-reported outcomes: results of the COSMIN study. J. Clin. Epidemiol..

[23] Chiarotto A, Deyo RA, Terwee CB, Boers M, Buchbinder R, Corbin TP (2015). Core outcome domains for clinical trials in non-specific low back pain. Eur. Spine J..

[24] Schull MJ, Guttmann A, Leaver CA, Vermeulen M, Hatcher CM, Rowe BH (2011). Prioritizing performance measurement for emergency department care: consensus on evidence-based quality of care indicators. Can J Emerg Med..

[25] International Society for Pharmacoeconomics and Outcomes Research: Pharmacoeconomic guidelines around the world.https://www.ispor.org/peguidelines/index.asp (2014). Accessed 14 Aug 2014

[26] Gold MR, Siegel JE, Russell LB, Weinstein MC (1996). Cost-effectiveness in health and medicine.

[27] Jones J, Hunter D (1995). Consensus methods for medical and health services research. BMJ.

[28] Walter, E., Zehetmayr, S.: Guidelines on health economic evaluation—consensus paper. Institute for Pharmaeconomic Research, Vienna (2006)

[29] Behmane, D., Lambot, K., Irs, A., Steikunas, N.: Baltic guideline for economic evaluation of pharmaceuticals (pharmacoeconomic analysis) (2002). https://www.ispor.org/PEguidelines/source/Baltic-PE-guideline.pdf

[30] Agency for Quality and Accreditation in Health Care, Department for Development, Research and Health Technology Assessment: The Croatian guideline for health technology assessment process and reporting. Agency for Quality and Accreditation in Health Care, Zagreb (2011)

[31] Kristensen, F.B., Sigmund, H. (eds.): Health technology assessment handbook Copenhagen: Danish Centre for Health Technology Assessment. National Board of Health (2007)

[32] Lääkkeiden Hintalautakunta Läkemedelsprisnämden: Preparing a health economic evaluation to be attached to the application for reimbursement status and wholesale price for a medicinal product. Ministry of Social Affairs and Health, Pharmaceuticals Pricing Board, Helsinki (2013)

[33] Haute Autorité de Santé: Choices in methods for economic evaluation. Haute Autorité de Santé, Saint-Denis La Plaine (2012)

[34] Szende Á, Mogyorósy Z, Muszbek N, Nagy J, Pallos G, Dózsa C (2002). Methodological guidelines for conducting economic evaluation of healthcare interventions in hungary: a hungarian proposal for methodology standards. Eur J Heal Econ..

[35] Ministry of Health of Hungary: Az emberi Eroforrások Minisztériuma szakmai irányelve az egészség-gazdaságtani elemzések készítéséhez (2013)

[36] Health Information and Quality Authority: Guidelines for the economic evaluation of health technologies in Ireland (2010)

[37] Capri S, Ceci A, Terranova L, Merlo F, Mantovani L (2001). Guidelines for economic evaluations in italy: recommendations from the Italian group of pharmacoeconomic studies. Drug Inf. J..

[38] López-Bastida J, Oliva J, Antoñanzas F, García-Altés A, Gisbert R, Mar J (2010). Spanish recommendations on economic evaluation of health technologies. Eur. J. Heal Econ..

[39] Pharmaceutical Benefits Board: General guidelines for economic evaluations from the Pharmaceutical Benefits Board (LFNAR 2003:2) (2003)

[40] National Institute for Health and Care Excellence: Guide to the methods of technology appraisal 2013 (2013) 27905712

[41] Rappange DR, Van Baal PH, van Excel NJ, Feenstra TL, Rutten FF, Brouwer WB (2008). Unrelated medical costs in life years gained: should they be included in economic evaluations of healthcare interventions?. PharmacoEconomics..

[42] Van Baal P, Meltzer D (2016). Future costs, fixed healthcare budgets, and the decision rules of cost-effectiveness analysis. Health Econ..

