# Supplementary material for: Consensus-based cross-European recommendations for the identification, measurement and valuation of costs in health economic evaluations: a European Delphi study
Source: Eur J Health Econ. 2017 Dec 19;19(7):993–1008. doi: 10.1007/s10198-017-0947-x (PMC6105226; doi:10.1007/s10198-017-0947-x)
Supplement: Supplementary file 1 — Supplementary material 1 (DOCX 24 kb) [file 10198_2017_947_MOESM1_ESM.docx]

**Appendix 1**. Overview of relevant cost categories and cost items to include in an economic evaluation in cross-European context.*

| **Cost categories** |
| --- |
| **1. Healthcare services** |
| Cost on: hospitalization; ICU; Emergency visits; medical specialist at an outpatient clinic; diagnostic services; medical devices; treatment procedures; day treatment in a hospital; medication; allied healthcare providers; mental healthcare services; preventive care; general practitioner visits; institutionalized care; palliative care; home care; supportive care; social care/welfare; respite care; complementary therapists; e-health |
| **2. Intervention costs** |
| Cost on: administration; planning; implementation; supervision and monitoring; training; donated items (such as drugs, vaccines, supplies or equipment) |
| **3. Patient and family costs** |
| Cost on: patient-out-of-pocket expenses; patient time; travel costs; informal caregivers time (not fully compensated); informal caregivers time (fully compensated) |
| **4. Lost productivity costs** |
| Cost on: absenteeism; reduced productivity while at work (i.e. presenteeism) |
| **5. Future costs** |
| Future healthcare costs incurred for disorders related and unrelated to the intervention |

*The nature of the disorder, intervention, and treatment under study determines the costs that should be included.

**Table 4.** Summary of consensus-based recommendations

| ***Component/topic*** | ***Recommendation*** | ***Arguments*** |
| --- | --- | --- |
| Perspective | Societal perspective | It is likely that relevant costs are missed when a narrower perspective is used, because often sectors other than healthcare may incur costs or costs savings as a result of the intervention |
| Identification of costs | Depending on the nature of the disorder, intervention, and treatment under study. Costs of healthcare services and social care services, intervention costs, patient and family costs, lost productivity costs, and future healthcare costs. | Relevant cost categories. |
| **Measurement of resource use** | |  |
| Healthcare services | Patient level data | Patient self-report methods are preferred over national databases, because not all services are covered in these databases |
| Patient-out-of-pocket expenses | Patient-reported expenses | Most reliable source to obtain these data |
| Patient time costs | Patient-reported time | Most reliable source to obtain these data |
| Travel costs | Standard distances | To avoid random differences between groups |
| Informal care costs | Self-report informal caregivers | Most reliable source to obtain these data |
| Absenteeism from paid labor | Self-reported sick leave due to the disease under study | Can be more accurately attributed to the disorder under study and may more easily be available than reports from many individual employers. |
| Presenteeism | Obtain ratings of both the quantity and quality of work performed in a standardized way. | Standardized method |
| **Valuation of resource use** | |  |
| Healthcare services | Country specific standard/unit costs | Representative for the situation in the country under study |
| Supportive care/ social care services | Country specific standard/unit costs | Representative for the situation in the country under study |
| Patient out-of-  Pocket expenses | Patient-reported costs | Large variations between patients. |
| Patient time/ informal care | National average wages of unskilled labor sex/age-specific |  |
| Travel costs | Use of standard distances between the patient’s home and the healthcare provider  Travel by public transport: tariffs  Travel by car: standard costs per kilometer/mile | Tariffs are closely related to market prices, and are expected to resemble opportunity costs adequately  Standard costs per kilometer/mile to avoid random differences between groups |
| Absenteeism | Friction cost approach.  National sex/age-specific average wages of the population as a whole | Human capital approach, an alternative, is expected to lead to overestimation of productivity losses |
| Presenteeism | National sex/age-specific average wages of the population as a whole |  |
| VAT | Include value added taxes (VAT) | Part of the true costs of healthcare. |
| Discounting | Country specific discounting rates Sensitivity analysis: lowest and highest European discounting rates | Representative for the situation in the country under study |
| Type of economic evaluation | Cost-Utility Analysis | Enables comparison across disorders and interventions. |
| Study design | Both a model-based approach and a trial-based approach are appropriate depending on the research question under study. |  |
